# Association between Daily Pattern of Physical Activity and Depression: A Systematic Review

**DOI:** 10.3390/ijerph19116505

**Published:** 2022-05-26

**Authors:** Vincenza Gianfredi, Pietro Ferrara, Flavia Pennisi, Giulia Casu, Andrea Amerio, Anna Odone, Daniele Nucci, Monica Dinu

**Affiliations:** 1 Department of Biomedical Sciences for Health, University of Milan, Via Pascal 36, 20133 Milan, Italy; vincenza.gianfredi@unimi.it; 2 CAPHRI Care and Public Health Research Institute, Maastricht University, 6200 MD Maastricht, The Netherlands; 3 Department of Public Health, Experimental and Forensic Medicine, University of Pavia, 27100 Pavia, Italy; pietro.ferrara@unipv.it (P.F.); anna.odone@unipv.it (A.O.); 4 Center for Public Health Research, University of Milan-Bicocca, 20900 Monza, Italy; 5 School of Medicine, Vita-Salute San Raffaele University, Via Olgettina 58, 20132 Milan, Italy; pennisi.flavia@hsr.it (F.P.); casu.giulia@hsr.it (G.C.); 6 Department of Neuroscience, Rehabilitation, Ophthalmology, Genetics, Maternal and Child Health (DINOGMI), Section of Psychiatry, University of Genoa, 16146 Genoa, Italy; andrea.amerio@unige.it; 7 IRCCS Ospedale Policlinico San Martino, 16132 Genoa, Italy; 8 Department of Psychiatry, Tufts University, Boston, MA 02155, USA; 9 Nutritional Support Unit, Veneto Institute of Oncology IOV-IRCCS, 35128 Padua, Italy; 10 Department of Experimental and Clinical Medicine, University of Florence, 50121 Florence, Italy; monica.dinu@unifi.it

**Keywords:** exercise, circadian rhythm, daily pattern, objectively measured physical activity, depression

## Abstract

Recent research suggested that daily pattern of physical activity (PA) may have an important association with depression, but findings are limited and contradictory. Our aim was to conduct a systematic review of the literature to summarize the literature evidence on the association between timing of PA and depression. A comprehensive search of PubMed/Medline and Scopus databases has been performed, and a total of five manuscripts have been thoroughly reviewed. The performed descriptive analysis shows lower levels of PA among individuals with depression or depressive symptoms, although evidence on the 24 h pattern of PA and depression is limited. An interesting finding is the association between lower PA during the morning, higher PA late in the evening (night), and depression or depressive symptoms. However, definitive conclusions could not be drawn due to the observational nature of the studies, their limited number, the high heterogeneity in the sample populations, and the studies’ differing outcome definitions and exposure assessments. Future studies considering not only the level of PA but also its daily variability might be important to further explore this novel area of research.

## 1. Introduction

Globally, an estimated 300 million people suffer from depression [1]. This condition is a leading cause of mental illness and a major public health issue, being responsible for low quality of life, self-harm, and premature mortality from suicide [1,2,3,4]. Depression is also linked with behavioral and biological features that affect physical health [5]. In fact, the burden associated with health loss due to depression has a negative impact on personal, community, and economic outcomes [6,7]. Therefore, effectively addressing this highly prevalent and disabling disorder would lead to a significant return on investment in terms of population well-being and economic outputs [6,7,8].

In recent decades, several potential determinants associated with higher risk of depression have been identified. They include modifiable lifestyle risk factors such as current smoking [9], at-risk alcohol consumption [10], overall nutritional inadequacy [11,12], social relationships [13], and physical inactivity [14,15]. Of note, the latter has a complex reciprocal relationship with depression, and a growing body of research investigates the effectiveness of preventive interventions that promote physical activity (PA) to reduce the prevalence and incidence of depressive disorders and symptoms [16]. In fact, many population-based studies have demonstrated a strong association between PA and a lower risk of clinically relevant depressive symptoms over time, as well as a reduction in the overall burden associated with depression [17,18]. These findings have been confirmed in a recently published systematic review and meta-analysis that considered objectively measured PA levels in order to provide better PA estimates and avoid possible relevant residual confounders associated with self-reporting [14].

Several possible physiological mechanisms through which PA helps with preventing and managing depression have been suggested, such as improved vascular function and oxygenation, regulation of the hypothalamic-pituitary-adrenal axis, noradrenergic and serotonergic effects, and production of neurotrophic factors [19,20,21,22,23]. More recently, some research has suggested that the timing of PA could be linked to depression [24,25], probably due to a shift in the circadian rhythm in depressed patients [26]. This shift leads to changes in physical, mental, and behavioral patterns during the 24-h cycle that result in a shift in preferred activity times [27,28]. Therefore, the PA timing could be a significant predictor of depressive symptoms and depression [24,25]. However, further research is needed to explore such a relationship and draw more robust conclusions. Hence, this systematic review aimed to summarize the current literature on the association between the time of day when PA is performed and depression/depressive symptoms.

## 2. Materials and Methods

The methods of this review were defined in advance; they follow the international guidelines for the proper conduct and reporting of systematic reviews: PRISMA (Prepared Items for Systematic Reviews and Meta-Analysis) [29]. A standardized protocol identifying the research question, the search strategy, and inclusion and exclusion criteria was developed and shared within the research team and fully approved before starting the review. The final version of the protocol was registered in PROSPERO, an international database of prospectively registered systematic review protocols (ID: CRD42021278924).

### 2.1. Search Strategy and Data Sources

Two investigators (GV and DN) retrieved studies by searching two main electronic databases, PubMed/Medline and Scopus, as they are both widely used resources in the biomedical field. Additionally, we consulted professionals involved in the field and analyzed reference lists of the included articles, with the aim of collecting other potentially relevant research output. The literature search was carried out in September 2021, and it was developed based on a combination of keywords related to depression (and similar) and hourly patterns of PA (and similar) by including both MeSH terms and free text words. Keywords were logically combined with the Boolean operators “AND”, “OR”, and “NOT”. The full search strategy is available in Supplementary Table S1.

### 2.2. Inclusion and Exclusion Criteria

A detailed description of inclusion and exclusion criteria, outlined according to the acronym PEOS (Population, Exposure, Outcome, Study design), is reported in Supplementary Table S2. We considered eligible studies those assessing adults (both sexes) with a clinical diagnosis of depression or depressive symptoms but no other medical conditions that reported incidence or prevalence of depression. PA had to be measured objectively by accelerometers, pedometers, or other means, and the hourly amount of PA performed during the day (24-h PA) had to be reported. Regarding the study design, only original epidemiological studies (case-control, cross-sectional, or cohort studies) were considered eligible. Conversely, studies assessing the association between depression and PA that was not objectively measured and not reported as a 24-hourly pattern (e.g., reported in aggregate form as total amount of daily PA) were excluded. We also excluded studies in which participants were women with a diagnosis of depression during pregnancy or postpartum. We made this decision because postpartum depression might be due to different etiological causes. Other restriction criteria were: the manuscript language had to be English and the full text available. Nonhuman studies (animal models), nonoriginal papers (e.g., reviews, book chapter, letters to the editor, brief notes, commentaries, conference papers) were also excluded. No publication date filter was applied.

### 2.3. Studies Selection and Data Extraction

The identified studies were independently analyzed by two authors (FP and GC) in a two-step process: first, manuscript titles and abstracts were analyzed to identify eligible articles; then, the second step consisted of reviewing the full manuscript text. In both phases, any disagreement between the reviewers was resolved through discussion until consensus was reached. If consensus could not be reached, a third senior author (VG) was consulted to resolve doubts. Article screening was performed manually and with the EndNote^®^ 9.0 software (Clarivate™, Philadelphia, PA, USA).

Data extraction was carried out independently by two authors (FP and GC), and divergences were resolved through discussion. A third author (VG) performed random checks. Extracted data were reported in a standardized spreadsheet (Excel for Windows) created specifically for data extraction. Extracted data included: full details of the references such as first author surname, year of publication, country and year in which the study was conducted, main characteristics of the subjects recruited in each study, device used for PA measurement, duration of PA measurement, diagnostic tool for depression, number of depressed subjects, main results, funding, and conflicts of interest.

### 2.4. Quality Assessment

The quality of the included studies was assessed using the Newcastle-Ottawa Quality Assessment Scale (NOS) [30]. The NOS is a validated tool developed to check the methodological quality of observational studies. The NOS provides a checklist for case-control and cohort studies. The NOS consists of 8 questions in 3 main domains: selection, comparability, and outcome. The final score ranges from 0 to 9. In agreement with previous research [31,32], studies were classified as high, moderate, or low quality when their NOS score was ≥7, 4–6, or ≤3, respectively.

### 2.5. Analysis and Results Presentation

The extracted data, as previously described [33,34], were used to report the main results obtained in tabular and synthetic form. The data collected, retrieved, and evaluated in this review were used to evaluate the possible association between timing of PA throughout the day and depression.

## 3. Results

### 3.1. Literature Search and Quality Evaluation

**Figure 1:**
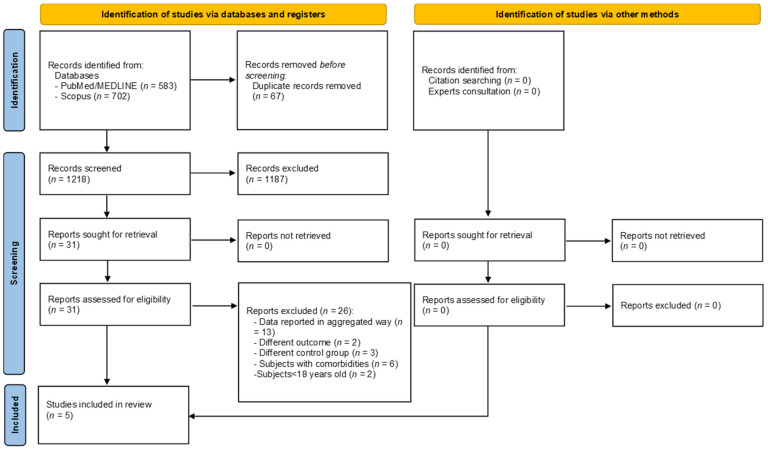
PRISMA flow diagram reporting the selection process.

A total of 1285 articles were retrieved, of which 583 were from PubMed/Medline and 702 from Scopus. After a preliminary screening, 67 articles were excluded because they were duplicates, and 1187 were excluded because they were not original papers (review, letter to editor, editorial, protocol, etc.) or because they focused on different topics. After the title and abstract analysis, a total of 31 articles were consulted in full, but 26 were excluded, as shown in Supplementary Table S3 [35,36,37,38,39,40,41,42,43,44,45,46,47,48,49,50,51,52,53,54,55,56,57,58,59,60]. At the end of the selection procedure, 5 articles were included in the systematic review [24,61,62,63,64]. Figure 1 depicts the selection process.

### 3.2. Main Characteristics of the Included Studies

Of the included studies, one was a prospective cohort study, while the others were case-control studies. Their characteristics are reported in Table 1. Most of them were conducted within the last 3 years [24,62,63], although the first study to objectively measure PA in relation to depression was published in 1985 [64]. Half of the studies were conducted in Europe [24,62,63]. The smallest sample size included 48 patients [64], and the largest sample size included 359 patients [62]. Two studies reported no attrition [61,64], meaning that none of the recruited participants left during the study. Conversely, the highest attrition registered was 1368 participants [63].

The studies included patients with an history of affective disorders recruited from hospital settings [61] and psychiatric research units [64], outpatients [24,62,63], and healthy controls [24,61,62,63,64]. All the studies used validated tools to diagnose depression-related outcomes. In particular, half of them used tools developed based on the Diagnostic and Statistical Manual of Mental Disorders, fourth edition (DSM-IV) [24,61,62]. Among the participants, the depressed patients ranged from 50% to 75% of the total. Considering the participants’ characteristics, depressed patients were mostly represented by women (4 studies out of 5) [24,61,62,64]. The mean age of the depressed patients ranged from 34 to 57 years, while that of the controls ranged from 39 to 57 years.

The PA was measured by accelerometer in all the analyzed studies. The most frequently reported (in 50% of the included studies) was the GENEActiv actigraphy device [24,62]. In 4 out of 5 studies, the device was worn on the wrist [24,61,62,64]. Of these, two studies [24,64] specified that the device was worn on the nondominant wrist, while in one study it was worn on the upper right arm [63]. In all studies, participants were asked to wear the device for at least 3 days; finally, in more than half of the studies, participants were instructed to wear the device day and night for 14 consecutive days [24,61,62].

### 3.3. Quality Assessment

According to the defined cut-points, all the articles were judged as high quality. Three studies scored 7 [61,62,64], and the scores for the remaining two totaled 8 points [24,59]. The main concerns were associated with the selection of controls (Item 3), which was not fully described in two studies, and the nonresponse rate (Item 8), which was not described in two other studies. The details are reported in Supplementary Table S4. Moreover, 80% of the studies included received funds [24,61,62,63]; in one study, the funding information was not specified [64]. Lastly, one study detailed a potential conflict of interest [61], three studies declared no conflict of interest [24,62,63], and in one study, it was not specified [64].

### 3.4. Main Results

Overall, more than half of the included studies reported a statistically significant association between lower PA and depression [24,62,64]. The presence (*p* = 0.05) and severity (*p* < 0.001) of depressive disorders were associated with a lower overall daily activity level compared with healthy controls. One of these papers specified that the level of activity was significantly lower from 7 a.m. to 10 p.m. [64]. Another study showed that higher levels of depressive symptoms were associated with a more nocturnal activity pattern (1:30 a.m.) [61]. The same article also found that a higher BMI was associated with higher activity levels from 3 to 5.30 a.m. and with lower activity levels from 10 a.m. to 10.30 p.m. [61]. Older age was associated with less activity during the day, evening, and night from 11 a.m. to 5.30 a.m. On the contrary, the authors found that in healthy controls, BMI and age only marginally affected activity patterns and only in a narrower portion of the 24 h period [61]. Older age and higher BMI were also confirmed to be associated with a lower overall daily activity by Difrancesco et al. [62]. The authors also found an association between the severity of depressive and anxiety symptoms and other sociodemographic factors such as higher education level, more chronic diseases, and smoking [62]. Interestingly, older age was associated with earlier morning activity and less activity in the late afternoon. These data suggest a stronger association between age and daily pattern of PA, whereas the presence and severity of depression were more associated with lower overall activity level but not with the daily pattern [62]. Finally, only one article found no differences in the 24 h activity between depressed and nondepressed subjects, even after excluding patients treated with antidepressant drugs [63].

## 4. Discussion

To the best of our knowledge, this is the first systematic review assessing the association between the daily pattern of PA and depression/depressive symptoms. Overall, a lower level of PA was found among individuals with depression or depressive symptoms, while evidence on the 24 h pattern of PA and depression was very limited. This review focused on 5 studies that reported hour-by-hour PA data by suggesting a possible link between PA timing and depression. Conversely, most of the studies on PA and depression reported data in an aggregate way, without giving insight into the temporal pattern, and thus, such papers were not considered for this literature review. However, the sample sizes of the analyzed manuscripts were small, and this could be one of the reasons why statistical significance was not reached in most cases. Furthermore, the direction of the association could not be defined due to the observational nature of the studies included.

In recent years, several studies have suggested that the timing of PA could be linked to depression due to a shift in the circadian rhythms of depressed patients [24,25,26]. In fact, the circadian rhythm, which plays an important role in regulating sleep/wake cycles, metabolism, hormone secretions, immune function, and cell cycle control, may be anticipated or delayed in depressed individuals with regard to activity times. This leads to changes in physical, mental, and behavioral patterns during the 24 h cycle [27,28]. Although the mechanisms responsible for circadian rhythm regulation are yet to be fully understood, oscillations of certain neurotransmitters in depression, including serotonin, norepinephrine, and dopamine may be involved in this association [28,65,66]. Another mechanism could be related to alterations in the circadian release of sleep-related hormones and metabolites, due to the disruption of the sleep/wake cycle [67]. This could result in a shift in preferred times for PA, with the latter being a predictor of depressive symptoms and depression [24,25]. Support for this hypothesis also comes from chronotype studies, which report a significant association between evening type and depression/depressive symptoms [68].

In our study, a considerable heterogeneity among studies was found, making it difficult to discern the potential role of PA patterns on depression. An interesting finding, however, was the association between lower level of PA during the morning, higher level of PA late in the evening (night), and depression or depressive symptoms. Greater levels of activity in the late evening between 10 p.m. and 1:00 a.m. were also found among borderline personality disorder patients with depressive symptoms compared with healthy controls [60]. These observations are in agreement with the theory that changes in the diurnal activity rhythm in depressed individuals may cause a shift in preferred PA times and an increase in activity level during the evening/night, resulting in altered sleep duration and less exposure to beneficial daylight. Additionally, anhedonia, a symptom of depression, could be responsible for the later start of PA in the morning among subjects with depression compared with healthy controls.

Although these findings are intriguing, the high heterogeneity observed in many aspects of the included studies might limit the interpretation and generalizability of our results: Depression and depressive symptoms were diagnosed using different tools or different scores, and both hospital-based patients and subjects from the community were included in the original studies. PA, meanwhile, was reported using different units of measurement, and although all but one [63] of the included studies used a wrist-worn accelerometer, this instrument was shown to be less sensitive and specific than the thigh-worn accelerometer [69]. Finally, almost all the retrieved studies were case-control studies, thus not allowing us to explore either temporality or causality. In addition, as PA was not measured over time, it was not possible to assess the risk of depression by differentiating between short- and long-term exposure to a certain daily pattern of PA.

### 4.1. Strengths and Limitations

Some strengths and limitations should be considered. First, this is a systematic review limited to only two databases. Optimal searches in systematic reviews could include other medical databases, for instance EMBASE, Web of Science, and Google Scholar [70]. However, evidence reports a high overlap (the range is between 64.6% to 95.8%) of records identifiable by searching different databases [71]. Moreover, the assessment of two databases is in line with the minimum requirements set by the PRISMA guidelines for systematic reviews. Second, we limited the search to articles published in English. However, no articles were removed because of this language limitation, so we are confident that our review is not biased by our selection process. Furthermore, English is the scientifically recognized language for publication. Third, the evidence on this topic is still scarce, with a very low number of included articles, and the level of heterogeneity is high. For this reason, it was not possible to carry out a meta-analysis, and the evidence we found allows only preliminary inferences to be drawn. In addition, we limited our search to only observational studies, excluding any type of trials. This might have limited the total number of included studies as well as the possibility of inferring etiological causes. Lastly, only half of the studies adopted psychometric scales based on DSM criteria, thus reducing the clinical and diagnostic homogeneity of the overall sample [72].

On the other hand, being the first systematic review on the topic, the article underlines the need for future research on this under-explored area. More knowledge on the relationship between PA timing and depression symptoms could be useful in both clinical and public health settings. Indeed, as changes in PA pattern could be related to changes in depressive symptoms and anxiety and/or to circadian rhythm alterations in depression [73], as well as to other forms of momentary assessments in depression [74], the combination of these measures could facilitate a new area of time-specific precision medicine to improve care.

### 4.2. Implications for Public Health Policies and Practice

With regard to public health and preventive strategies, this study suggests that lower overall PA level is associated with higher risk of depression and, despite the above-mentioned limitations, that timing may also be important when it comes to depression. These factors are strongly influenced by sociocultural aspects and the environment. Indeed, cities with a high density of bicycle and pedestrian paths can induce more active lifestyles [75]. At the same time, urban green space is also associated with more PA performed, which in turn is associated with physical and mental health [76]. Moreover, a recent systematic review revealed that urban green space is associated with a higher level of recreational activities performed during the day and, consequently, a lower level of distress and depressive symptoms [76]. Although data on leisure activities and working hours are not reported in the included primary studies, it can be assumed that most people work on weekdays and through the afternoon. Therefore, decreasing the time spent in sedentary activities during the day and promoting PA sessions during evenings and weekend days, with both formal PA and leisure time, may be a cost-effective nonpharmacological treatment for individuals with depressive symptoms.

On the basis of the evidence gathered so far, it can be argued that primary prevention interventions, such as encouraging standing or stepping during working hours, especially for white-collar workers, may be useful for improving the total amount of PA, for reducing sedentary time during the morning hours, and for reducing the short- and long-term risk of burn-out and late-life depression. These primary prevention interventions aimed at promoting healthy lifestyles could also have some secondary prevention effects. Indeed, PA practiced by individuals with depression or depressive symptoms reduces the risk of depression relapse and could improve treatment.

Regarding public policies, our data contribute to the exploration of the role of PA in depression. As depression is a major cause of disability globally [77], with a high risk of premature mortality and an approximately 10 years reduction in life expectancy [78], all countries should implement health policies to address this high-cost disease. For this reason, solid evidence on the impact of PA timing on depression needs to be collected and then implemented in public health.

## 5. Conclusions

In conclusion, our systematic review explored a new area of research that deserves further investigation. Our findings are derived from observational evidence and suggest that an overall lower level of PA is associated with a higher risk of depression. When timing was considered, most studies did not reach statistical significance or observed only marginal differences. Specifically, a lower level of PA early in the morning and a higher level of activity late in the evening/night were found to be associated with a higher occurrence of depression/depressive symptoms. Given the nature of the studies and their very limited number, the directions of these associations cannot be understood at present. However, these data highlight the importance of conducting further studies on this topic. Observational studies and trials targeting the appropriate timing and levels of PA could be important in reducing the high burden of depression in our society.

## Figures and Tables

**Table 1:** The main characteristics of the included studies, reported in alphabetical order.

Author, Year [Ref.]	County	Study Period	Study Design	Main Characteristics of the Sample	Sample Size (% of F); Age as Mean ± SD	Attrition	Device Used to Measure PA	Duration of PA Measurement	Validated Diagnostic Depression Tool	No. of Depressed Subjects	Main Results	Funds	CoI
Banihashemi, N.; 2016 [61]	Australia	NA	C-C	Hospital-based patients with history of affective disorders	168 cases (59.7 F); 35.7 ± 20.8 y 68 controls (64.7); 38.8 ± 21.4 y	0	Actiwatch-64/L/2/Spectrum, Philips Respironics, USA	5–22 days (mean = 14 days)	HDRS (based on DSM-IV)	118	Higher HDRS scores were associated with higher activity around 1:30 a.m.	yes	yes
Difrancesco, S.; 2021 [62]	The Netherlands	Recruitment 2004–2007; 9 y FU	Co	Participants with current (*n* = 93), remitted (*n* = 176), or no (*n* = 90) depression	359 (62% F); 50.1 ± 11.1 y	25	Wrist-worn GENEActiv device (Activinsights Ltd.), UK	14 consecutive days	CIDI and IDS (based on DSM-IV)	93 current and 176 remittent depressive subjects	The presence (*p* = 0.05) and severity (*p* < 0.001) of depressive and anxiety disorders were associated with a lower overall daily activity level but not with the timing of activity	yes	none
Lorenz, N.J.; 2019 [63]	Germany	NA	C-C	121 participants with pronounced depressive symptoms 121 matched nondepressed controls	242 (24% F); 56.52 ± 9.96	1368	SenseWear^®^ Pro 3 actigraph (BodyMedia Inc.; Pittsburgh, Pennsylvania)	7 consecutive days (on average 23.03 h of data was available per day)	CES-D	121	No differences were found in the 24 h activity between depressed and nondepressed subjects even after excluding patients treated with antidepressant drugs	yes	none
Minaeva, O.; 2020 [24]	The Netherlands	2020	C-C	58 adults with a depression in the past 6 months, 43 adults with acute depression in the past 1 month controls (*n* = 63)	121 (63.6); 52.13 ± 11.3	263	Wrist-worn GENEActiv device (Activinsights Ltd.)	14 consecutive days of all-day	CIDI, and IDS (based on DSM-IV)	111	Depressed subjects (acute: diagnosis less than 1 month before and chronic: more than 6 months) showed less PA than healthy controls in total during the 24 h; however, only marginally significant differences were detected later in the evening	yes	none
Wolff, E. A.;1985 [64]	USA (Bethesda)	1984	C-C	Patients with affective illness and a group of normal volunteers, all resident in an inpatient psychiatric research unit of the National Institute of Mental Health, Bethesda	30 cases (63% F); 37.9 ± 13.0 y 18 controls (20% F); 37.9 ± 13.0	0	Small, self-contained, solid-state, non-telemetric electronic device worn on the nondominant wrist	A minimum of 3 days	BHS	23	Depressed subjects showed significantly lower motor activity from 7 a.m. to 10 p.m. than healthy controls	NA	NA

Bunney-Hamburg scale; CIDI: Composite International Diagnostic; CES-D: Center for Epidemiologic Studies Depression Scale; Co: cohort study; CoI: Conflict of interest; F: female; FU: follow-up; HDRS: Hamilton Depression Rating Scale; IDS: Interview Depressive Symptomatology; NA: not available; QIDS: Quick Inventory of Depressive Symptomatology; SD: standard deviation; USA: United States of America; y: years.

## Data Availability

All data are published in the current manuscript or Supplementary Materials.

## Supplementary Materials

The following supporting information can be downloaded at: https://www.mdpi.com/article/10.3390/ijerph19116505/s1, Table S1. Search strategy in PubMed/MEDLINE and Scopus. Table S2. Detailed description of inclusion/exclusion criteria according to a Population, Exposure, Outcomes and Study design (PEOS). Table S3. Articles assessed in full and excluded with reasons. Table S4. The Newcastle-Ottawa Scale (NOS) quality assessment of the included studies, in alphabetical order.
