# Supplementary material for: Association between Daily Pattern of Physical Activity and Depression: A Systematic Review
Source: Int J Environ Res Public Health. 2022 May 26;19(11):6505. doi: 10.3390/ijerph19116505 (PMC9180107; doi:10.3390/ijerph19116505)
Supplement: Supplementary file 1 [file ijerph-19-06505-s001.zip › ijerph-1683109-supplementary.pdf]

Supplementary Table S1. Search strategy in PubMed/MEDLINE and Scopus.

SET	PubMed/MEDLINE
1	"Patient Health Questionnaire"[Mesh]
2	"Depressive Disorder"[Mesh]
3	“Depression"[Mesh]
4	depression[Title/Abstract]
5	depressive[Title/Abstract]
6	PHQ[Title/Abstract]
7	Sets 1-6 were combined with “OR”
8	"Screen Time"[Mesh]
9	"Exercise"[Mesh]
10	"Sports"[Mesh]
11	"Leisure Activities"[Mesh]
12	"Sitting Position"[Mesh]
13	"Supine Position"[Mesh]
14	"Sedentary Behavior"[Mesh]
15	“sedentary time” [Title/Abstract]
16	“sedentary pattern” [Title/Abstract]
17	“sedentary behaviour” [Title/Abstract]
18	“sedentary behavior” [Title/Abstract]
19	"Walking"[Mesh]
20	"Walking Speed"[Mesh]
21	"Bicycling"[Mesh]
22	"Endurance Training"[Mesh]
23	"Movement"[Mesh]
24	Movement*[Title/Abstract]
25	sport*[Title/Abstract]
26	physical activity[Title/Abstract]
27	physical activities[Title/Abstract]
28	physical exercise[Title/Abstract]
29	physical exercises[Title/Abstract]
30	walking[Title/Abstract]
31	stepping[Title/Abstract]
32	sitting[Title/Abstract]
33	Sets 8-32 were combined with “OR”
34	Set 7-33 were combined with “AND”
35	"Actigraphy"[Mesh]
36	"Accelerometry"[Mesh]
37	pedometer*[Title/Abstract]
38	ActivPAL [Title/Abstract]
39	24 hours [Title/Abstract]
40	24 hour [Title/Abstract]
41	24-hour [Title/Abstract]
42	24-hours[Title/Abstract]
43	“Diurnal patterns” [Title/Abstract]
44	“Diurnal pattern” [Title/Abstract]
45	Diurnal [Title/Abstract]
46	Hourly pattern [Title/Abstract]
47	Hourly patterns [Title/Abstract]
48	“Daily pattern”[Title/Abstract]

49	“Daily patterns”[Title/Abstract]
50	Chronotype [Title/Abstract]
51	Morningness [Title/Abstract]
52	Eveningness [Title/Abstract]
53	"Circadian Clocks"[Mesh]
54	"Circadian Rhythm"[Mesh]
55	Set 35-54 were combined with “OR”
56	Set 33-55 were combined with “AND”
57	"Review" [Publication Type]
58	"Review Literature as Topic"[Mesh]
59	review[Title/Abstract]
60	"Meta-Analysis" [Publication Type]
61	"Meta-Analysis as Topic"[Mesh]
62	"Network Meta-Analysis"[Mesh]
63	"Infant, Newborn"[Mesh]
64	"Infant"[Mesh]
65	"Child"[Mesh]
66	child[Title/Abstract]
67	children[Title/Abstract]
68	infant*[Title/Abstract]))
69	"Depression, Postpartum"[Mesh]
70	Sets 57-69 were combined with “OR”
71	Sets 7 and 56 were combined with “AND”
72	Sets 71 and 70 were combined with “NOT”
73	Set 72 was limited to English language

SET	Scopus
1	" Screen Time”[TITLE-ABS-KEY]
2	"Exercise"[TITLE-ABS-KEY]
3	Sport*[TITLE-ABS-KEY]
4	“Leisure activities”[TITLE-ABS-KEY]
5	“Sitting position”[TITLE-ABS-KEY]
6	“Supine position”[TITLE-ABS-KEY]
7	“Sedentary behaviour” [TITLE-ABS-KEY]
8	“Sedentary time” [TITLE-ABS-KEY]
9	“Sedentary pattern” [TITLE-ABS-KEY]
10	“Walking” [TITLE-ABS-KEY]
11	“Walking speed” [TITLE-ABS-KEY]
12	“Bicycling” [TITLE-ABS-KEY]
13	“Endurance training” [TITLE-ABS-KEY]
14	Movement*[TITLE-ABS-KEY]
15	“Physical activity” [TITLE-ABS-KEY]
16	“Physical activities” [TITLE-ABS-KEY]
17	“Physical exercise” [TITLE-ABS-KEY]
18	“Physical exercises” [TITLE-ABS-KEY]
19	“Stepping” [TITLE-ABS-KEY]
20	“Sitting” [TITLE-ABS-KEY]
21	Sets 1-20 were combined with “OR”

22	"Actigraphy"[TITLE-ABS-KEY]
23	"Accelerometry"[TITLE-ABS-KEY]
24	pedometer*[TITLE-ABS-KEY]
25	ActivPAL [TITLE-ABS-KEY]
26	24 hours [TITLE-ABS-KEY]
27	24 hour [TITLE-ABS-KEY]
28	24-hour [TITLE-ABS-KEY]
29	24-hours[TITLE-ABS-KEY]
30	“Diurnal patterns” [TITLE-ABS-KEY]
31	“Diurnal pattern” [TITLE-ABS-KEY]
32	“Diurnal”[TITLE-ABS-KEY]
33	“Daily patterns”[TITLE-ABS-KEY]
34	“Daily pattern”[TITLE-ABS-KEY]
35	Chronotype [TITLE-ABS-KEY]
36	Morningness [TITLE-ABS-KEY]
37	Eveningness [TITLE-ABS-KEY]
38	"Circadian Clocks” [TITLE-ABS-KEY]
39	"Circadian Rhythm” [TITLE-ABS-KEY]
40	Hourly pattern [TITLE-ABS-KEY]
41	Hourly patterns [TITLE-ABS-KEY]
42	Set 22-41 were combined with “OR”
43	Set 21-42 were combined with “AND”
44	“Patient health questionnaire”[TITLE-ABS-KEY]
45	“Depressive disorder”[TITLE-ABS-KEY]
46	“Depression”[TITLE-ABS-KEY]
47	“Depressive”[TITLE-ABS-KEY]
48	“PHQ”[TITLE-ABS-KEY]
49	
50	Set 44-49 were combined with “OR”
51	Set 43-50 were combined with “AND”
52	"Review”[Doctype]
53	“Book chapter”[Doctype]
54	“Conference paper”[Doctype]
55	“Letter”[Doctype]
56	“Note”[Doctype]
57	“Short Survey”[Doctype]
58	“Conference Review”[Doctype]
59	“Editorial”[Doctype]
60	Set 52-59 were combined with “OR”
61	Set 21-51 were combined with “AND”
62	Sets 60 and 61 were combined with “NOT”
63	Set 62 was limited to English language

Supplementary Table S2. Detailed description of inclusion/exclusion criteria according to a Population, Exposure, Outcomes and Study design (PEOS).

INCLUSION CRITERIA	
P = population	Adults (≥ 18 years old) with a clinical diagnosis of depression but no other medical conditions
E = exposure	Physical activity measured objectively by accelerometers, pedometers, or other means, reporting the hourly amount of physical activity performed during the day (24-hour physical activity pattern)
O = outcome	Incidence or prevalence of depression
S = study design	Original epidemiological studies (case-control, cross-sectional or cohort studies)
EXCLUSION CRITERIA	
P = population	Subjects under the age of 18, subjects with a clinical diagnosis of depression and additional medical conditions
E = exposure	Physical activity not measured objectively or reported as total amount per day instead of hour by hour during the day
O = outcome	Other psychological disorders
S = study design	Review article, systematic review, meta-analysis, trial, expert opinion, commentary, article with no quantitative information or details

Supplementary Table S3. Articles assessed in full and excluded with reasons

**Reason of exclusion**	**n. of studies**	**Author, year [Ref]**
Data reported in aggregated way	13	Asai et al., 2018; Del Pozo Cruz et al., 2020; Difrancesco et al., 2019; Dillon et al., 2018; Figueroa et al., 2021; Gonzalez et al., 2014; Helgadottir et al., 2015; Krane-Gartiser, Henriksen et al. 2014, Krane-Gartiser, Henriksen et al. 2015, Kuhs and Reschke, 1992; Mendlowicz et al., 1999; Todder et al., 2009; Volkers et al., 2003 [35-47]
Different control group	3	Foster et al. 1975, Benoit et al. 1985, Krane-Gartiser et al. 2017 [48-50]
Different outcome	2	Korszun et al. 2002, Hori et al. 2016 [51, 52]
Subjects with other comorbidities	6	Appelhans et al., 2012; Krane-Gartiser et al., 2016; Parker et al., 2002; Porta et al., 2018; Verkooijen et al., 2017; McGowan et al., 2019 [53-57]
Subjects younger than 18 years old	2	Finazzi et al., 2010; Merikanto et al., 2017[58-59]

Supplementary Table S4. The Newcastle-Ottawa Scale (NOS) quality assessment of the included studies, in alphabetical order.

Author, year [Ref]	Study type	Selection				Comparability		Outcome/Exposure^			Total
		Item 1	Item 2	Item 3	Item 4	Item 5a	Item 5b	Item 6	Item 7	Item 8	
Banihashemi, N. et al, 2016 [61]	Case control	*	*	-	*	*	*	*	*	-	7
Difrancesco, S. et al, 2021 [62]	Cohort study	*	*	*	-	*	*	*	-	*	7
Lorenz, N. et al, 2019 [63]	Case-control	*	*	*	*	*	*	*	*	-	8
Minaeva, O. et al, 2020 [24]	Case-control	*	*	*	*	*	*	*	*	*	8
Wolff, E. et al, 1985 [64]	Case control	*	*	-	*	*	*	*	*	*	7

^based on study design. Outcome was considered in cross-sectional and cohort studies, exposure was considered in case-control studies.

* Each star represents a high-quality criterion accomplished by the study

- represents that the study did not satisfy the criterion
